# Supplementary material for: Ultrathin positively charged electrode skin for durable anion-intercalation battery chemistries
Source: Nat Commun. 2023 Feb 10;14:760. doi: 10.1038/s41467-023-36384-5 (PMC9918723; doi:10.1038/s41467-023-36384-5)
Supplement: Supplementary file 1 — Supplementary Information [file 41467_2023_36384_MOESM1_ESM.pdf]

Supplementary Information

Ultrathin Positively Charged Electrode Skin for Durable Anion-Intercalation Battery Chemistries

Davood Sabaghi[1, 11], Zhiyong Wang[1, 2, 11], Preeti Bhauriyal[3, 11], Qiongqiong Lu[4], Ahiud Morag[1], Daria Mikhailovia[4], Payam Hashemi[1,2], Dongqi Li[1], Christof Neumann[5], Zhongquan Liao[6], Anna Maria Dominic[1], Ali Shaygan Nia[1,2], Renhao Dong[1, 7, *], Ehrenfried Zschech[8], Andrey Turchanin[5], Thomas Heine[3, 9, 10], Minghao Yu[1, *], Xinliang Feng[1, 2, *]

1. *Center for Advancing Electronics Dresden (cfaed) & Faculty of Chemistry and Food Chemistry, Technische Universität Dresden, Mommsenstraße 4, 01062 Dresden, Germany*
E-mail: renhaodong@sdu.edu.cn; minghao.yu@tu-dresden.de; xinliang.feng@tu-dresden.de
2. *Max Planck Institute of Microstructure Physics, D-06120 Halle (Saale), Germany*
3. *Theoretical Chemistry, Technische Universität Dresden, 01062 Dresden, Germany*
4. *Leibniz Institute for Solid State and Materials Research (IFW) e. V., 01069 Dresden, Germany*
5. *Institute of Physical Chemistry, Friedrich Schiller University Jena, 07743, Jena, Germany*
6. *Fraunhofer Institute for Ceramic Technologies and Systems (IKTS), Dresden 01109, Germany*
7. *Key Laboratory of Colloid and Interface Chemistry of the Ministry of Education, School of Chemistry and Chemical Engineering, Shandong University, Jinan 250100, China*
8. *Faculty of Chemistry, University of Warsaw, ul. Żwirki i Wigury 101, Warsaw 02-089, Poland*
9. *Institute of Resource Ecology, Helmholtz-Zentrum Dresden-Rossendorf, Leipzig Research Branch, 04316 Leipzig, Germany*
10. *Department of Chemistry, Yonsei University, Seodaemun-gu, Seoul 120-749, Korea*
11. *These authors contributed equally: Davood Sabaghi, Zhiyong Wang, Preeti Bhauriyal*

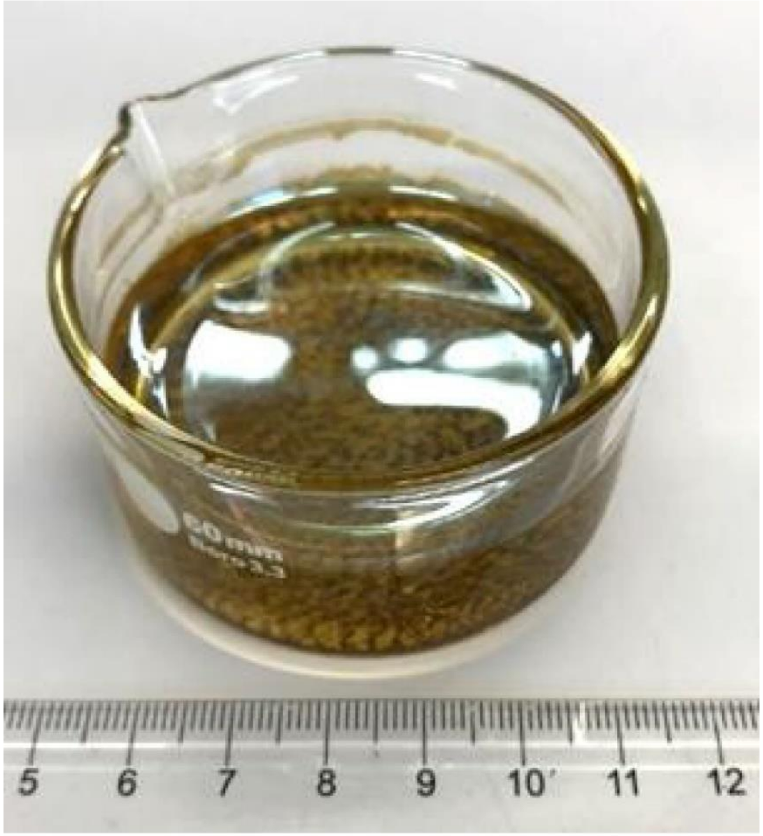

Supplementary Figure 1. Digital photo showing the as-obtained C2DP on the water surface.

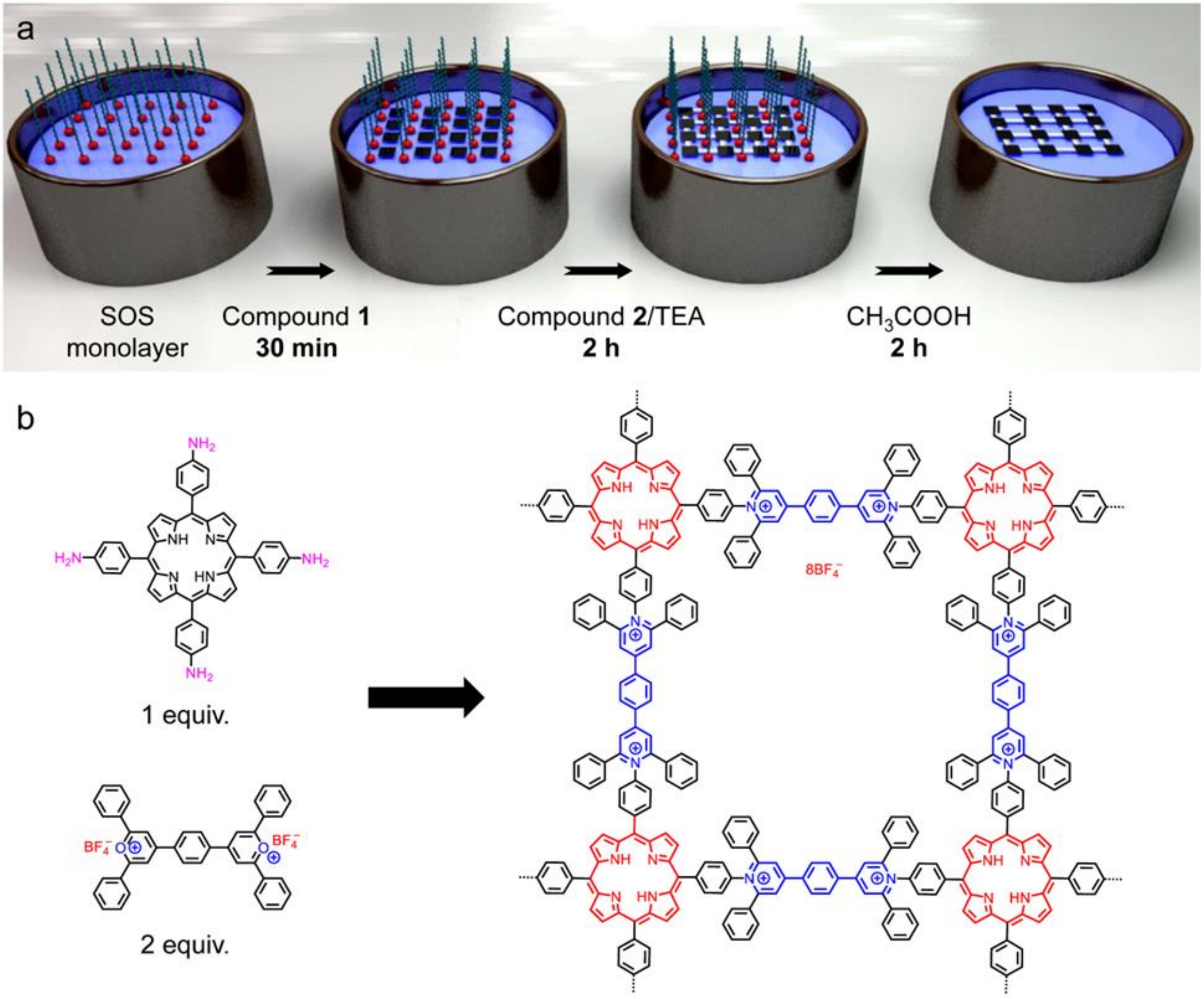

Supplementary Figure 2. a Schematic and **b** atomic illustration showing the synthetic route of C2DP.

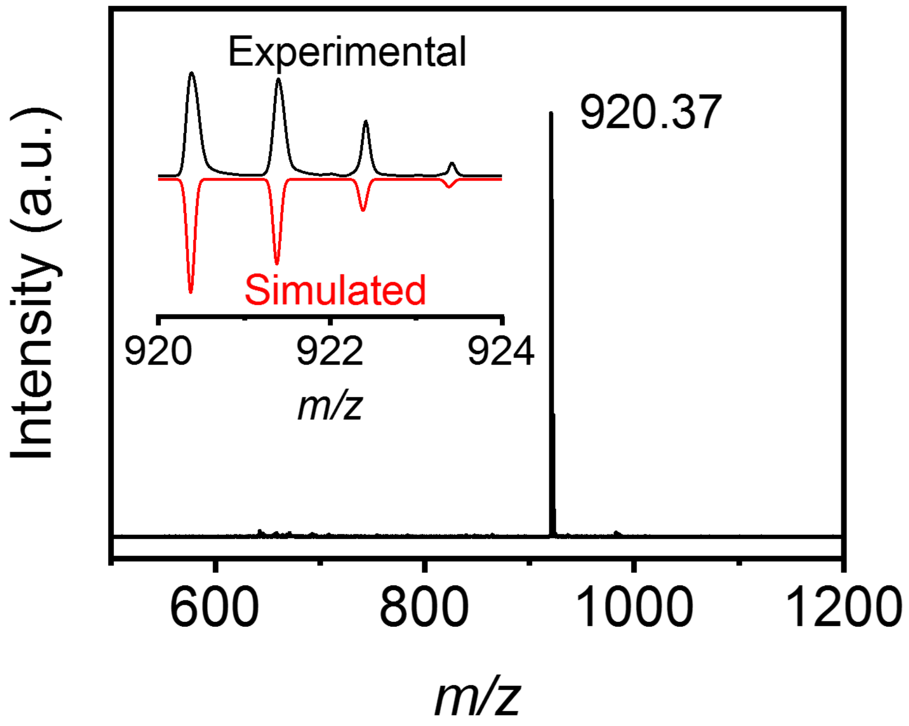

Supplementary Figure 3. MALDI-TOF MS analysis of the model reaction on the water surface.

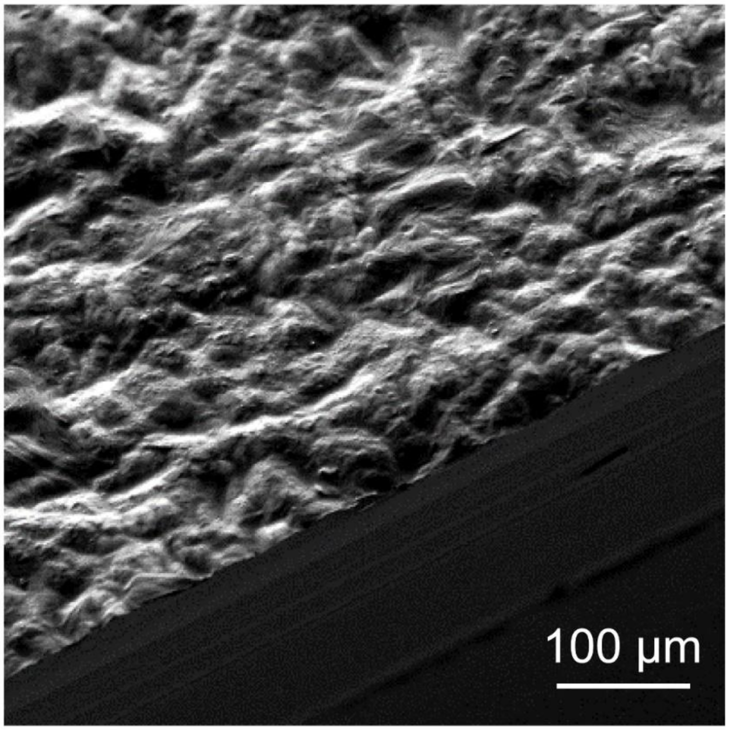

Supplementary Figure 4. SEM image of C2DP on a Si wafer.

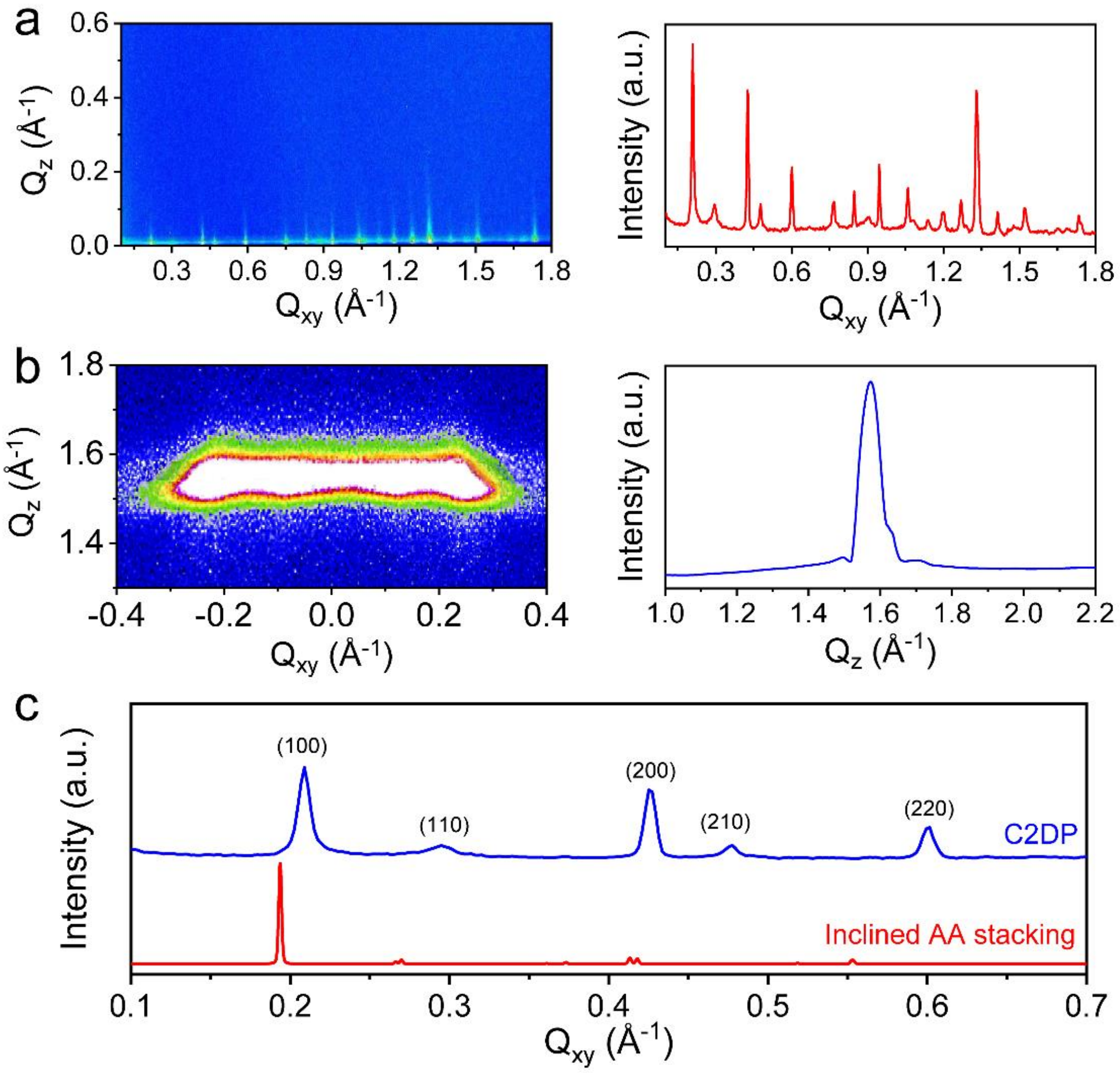

Supplementary Figure 5. a In-plane (near $Q_z = 0$) and **b** out-of-plan grazing incidence wide-angle X-ray scattering (GIWAXS) patterns and projections of C2DP. **c** In-plane projection of C2DP compared with that of the simulated inclined AA-stacking model.

The in-plane peaks at $Q_{xy} = 0.21$ and 0.42 Å^{-1}correspond to the (100) and (200) Bragg reflections of a square lattice with a = b = 30.5 Å. The measured lattice parameter is in excellent agreement with the structural model. Moreover, the crystallinity of C2DP is evidenced by the in-plane GIWAXS profile (Supplementary Figure 4a), which shows sharp and discrete Bragg spots near Q_Z = 0. In the out-of-plane direction (Supplementary Figure 4b), an intense arc at 1.58 Å^{-1} indicates the face-on orientation of layered C2DP and the presence of quasi-1D nanochannels (diameter of 30.5 Å) in C2DP perpendicular to the substrate. The resolved intensity profile agrees well with the calculated pattern of the AA-inclined stacking model (Supplementary Figure 4c), which also reflects the presence of quasi-1D nanochannels (diameter of 30.5 Å) in the C2DP membrane perpendicular to the substrate.

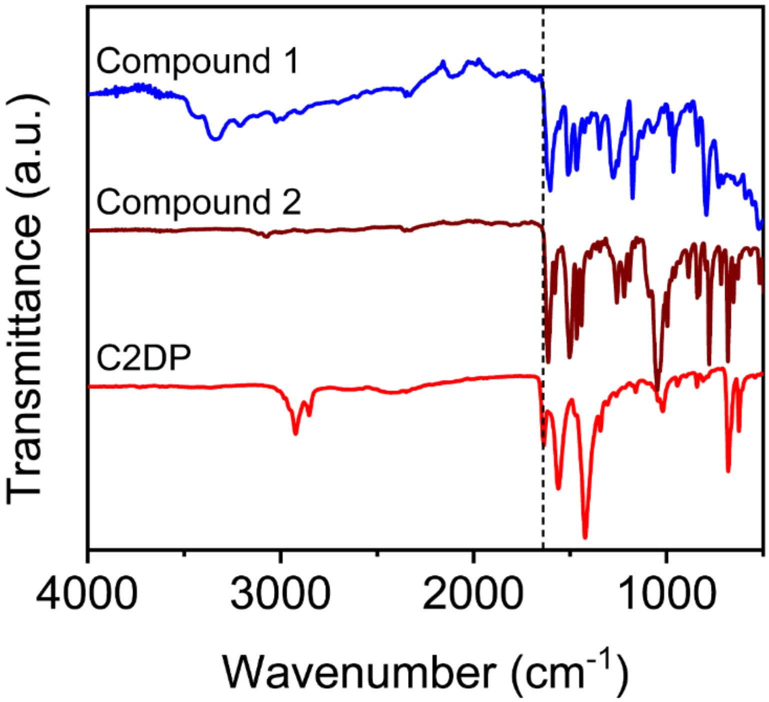

Supplementary Figure 6. FTIR spectra of compound **1**, compound **2**, and C2DP.

The efficient conversion of the amino groups in compound **1** to the positively charged pyridinium rings of C2DP was confirmed by the FTIR spectrum of C2DP with the appearance of the C-N^+ band at 1,638 cm^{-1}.

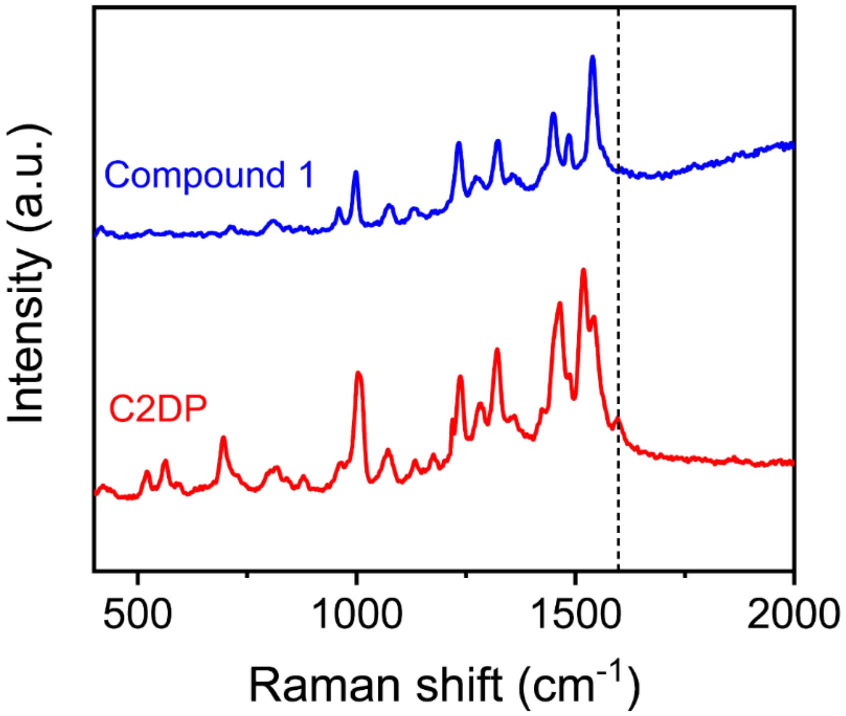

Supplementary Figure 7. Raman spectra of compound **1** and C2DP. Raman spectrum of C2DP displays a new peak at 1,595 cm^{-1}, which also verifies the formation of $C–N^{+}$ bonds.

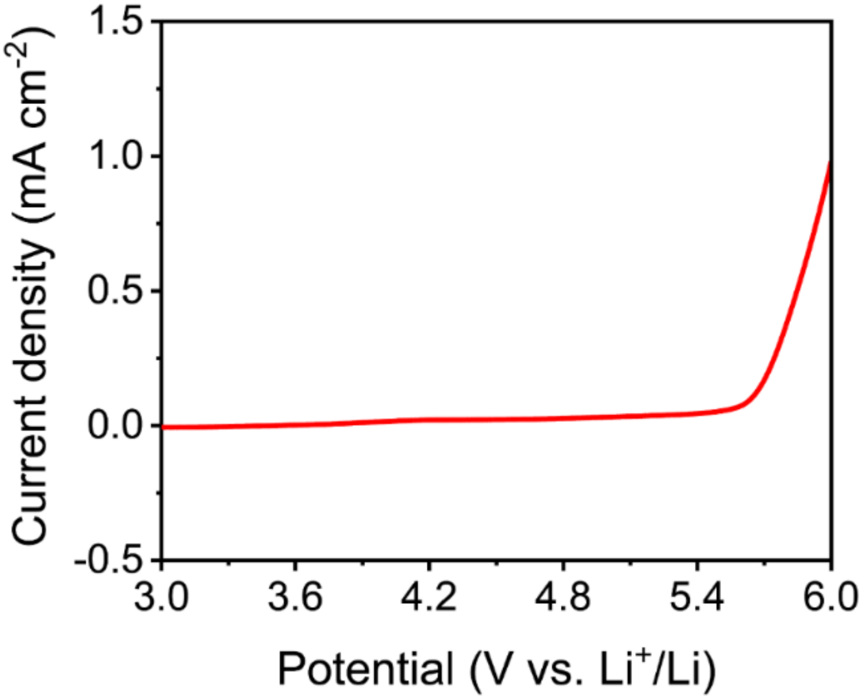

Supplementary Figure 8. Linear sweep voltammetry curve of C2DP loaded on the stainless steel substrate in 2 M $LiPF_6$.

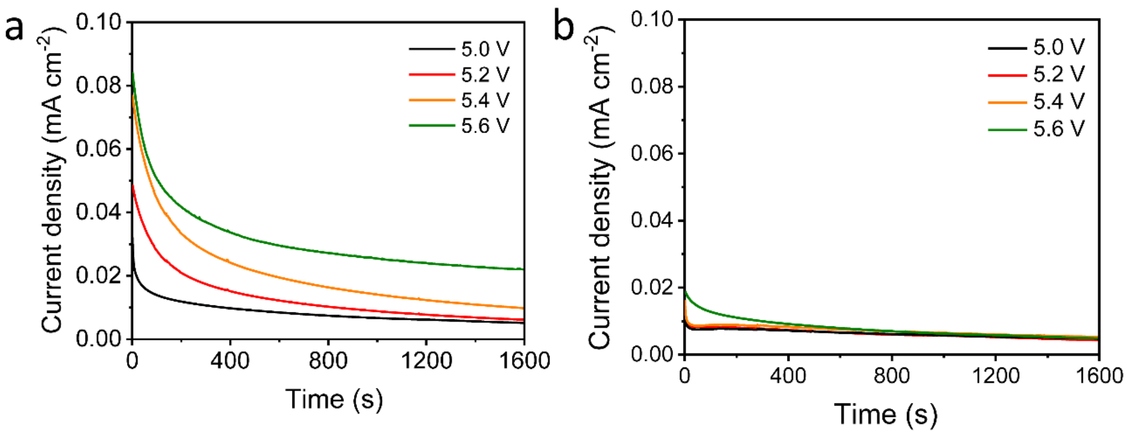

Supplementary Figure 9. The floating voltage test of **a** SS and **b** C2DP-SS with the chronoamperometry method at different voltages.

We carried out the floating voltage test of the stainless-steel current collector (denoted SS) and C2DP-loaded SS (denoted C2DP-SS) with the chronoamperometry method in 2 M $LiPF_6$ dissolved in DMC (Supplementary Figure 9). Four different voltages were employed, *i.e.*, 5.0 V, 5.2 V, 5.4 V, and 5.6 V. For SS, the leaking current density at 5.0 V was only 0.013 mA cm^{-2}, while it increased to 0.014 mA cm^{-2}, 0.018 mA cm^{-2} and 0.026 mA cm^{-2} at 5.2 V, 5.4 V, and 5.6 V, respectively. This result indicates the pronounced electrolyte decomposition reaction on SS at 5.4 V and 5.6 V. By contrast, C2DP-SS behaved almost the same at 5.0 V, 5.2 V, and 5.4 V, showing the fast current relaxation (< 11 s) and the low leaking current density of 0.0052 mA cm^{-2}. This result indicates that C2DP can completely inhibit electrolyte decomposition at 5.4 V owing to its electron-insulating property. When the voltage reached 5.6 V, although C2DP-SS showed a longer current relaxation time (300 s), the final leaking current is still around 0.005 mA cm^{-2}. This observation implies that C2DP can obviously suppress electrolyte decomposition even at 5.6 V.

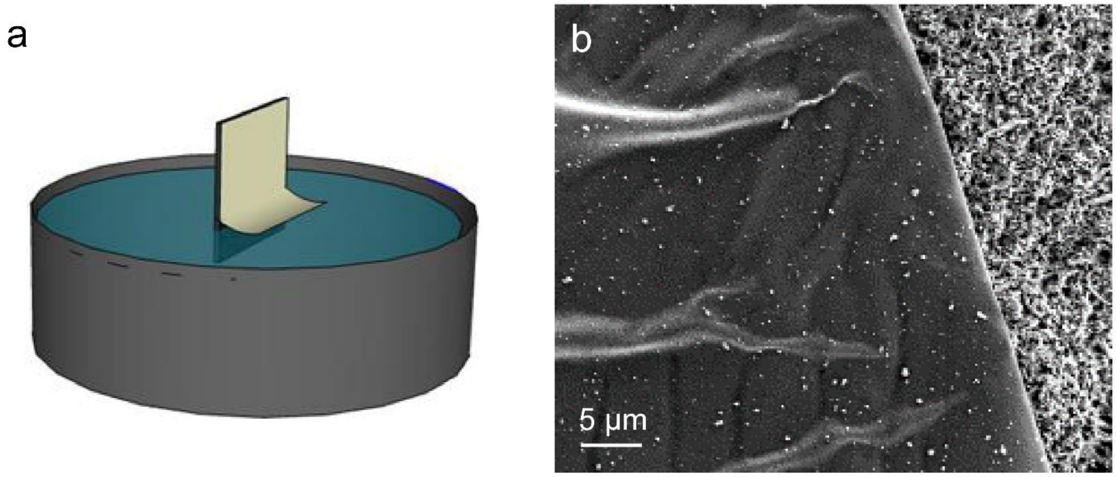

Supplementary Figure 10. a Schematic illustration showing the ‘fishing’ approach for transferring C2DP on the as-prepared graphite electrode. **b** SEM image of C2DP loaded on a graphite electrode.

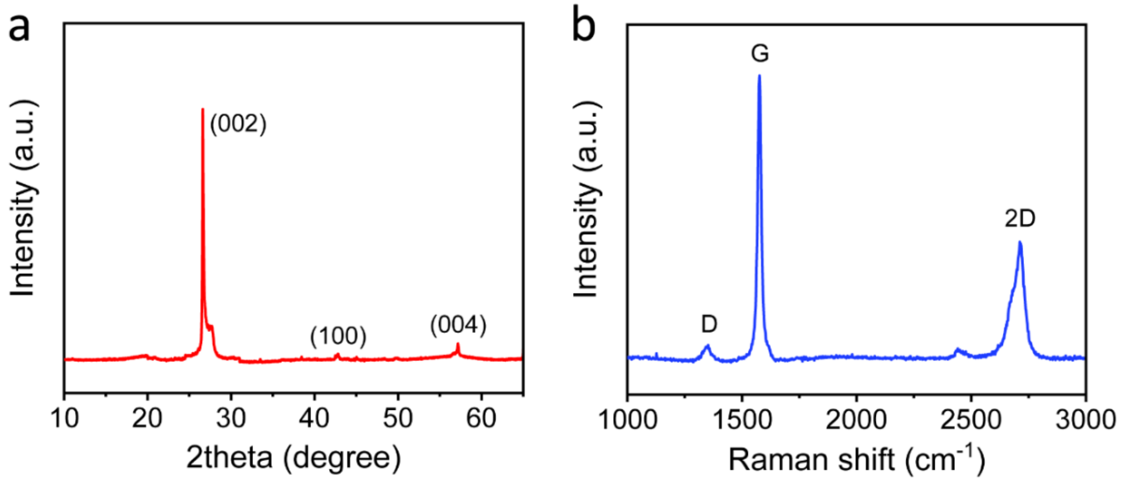

Supplementary Figure 11. a XRD and **b** Raman spectra of graphite.

XRD and Raman spectra of graphite were collected (Supplementary Figure 11). The sharp characteristic XRD peaks at 26.7° for the (002) plane and 57.3° for the (004) plane indicate the high crystallinity of graphite with an interlayer spacing of 0.336 nm. In the Raman spectrum, graphite shows three prominent characteristic peaks, namely the D band, the G band, and the 2D band. The sharp G band indicates the highly ordered graphite layers arranged in AB Bernal stacking. These characteristic Raman peaks again evidence the high crystallinity with rare carbon defects.

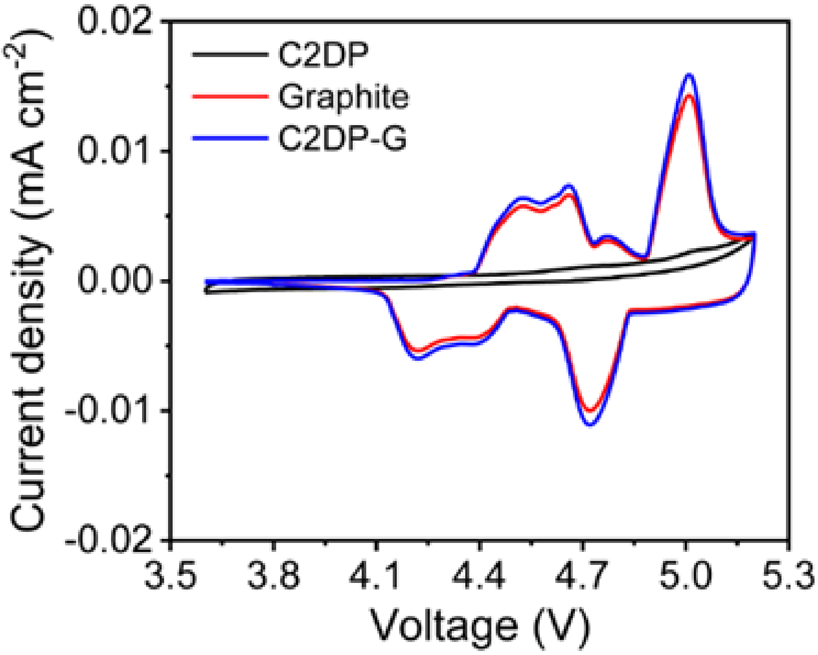

Supplementary Figure 12. CV curves of C2DP, the graphite electrode, and the C2DP-G electrode at 1 mV s^{-1}.

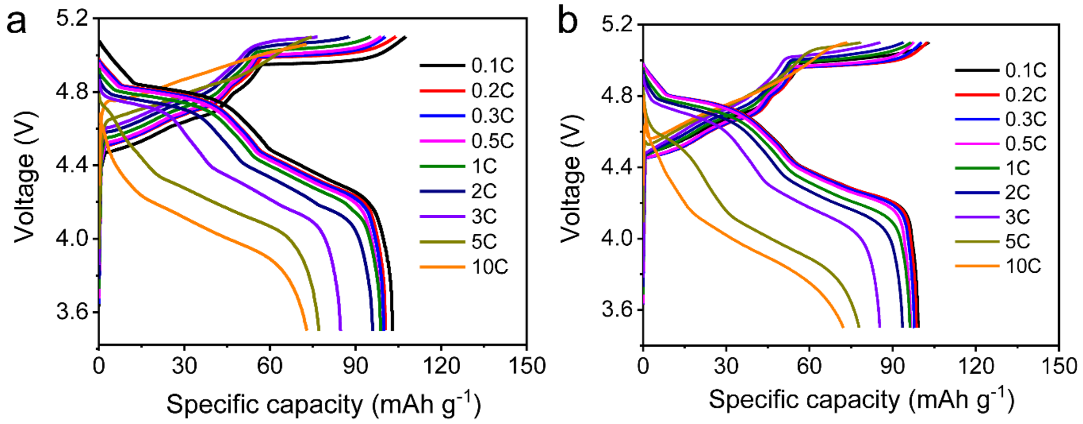

Supplementary Figure 13. GCD curves of the **a** graphite and **b** C2DP-G electrodes at different rates.

We defined 100 mA g^{-1} as 1 C, because the theoretical specific capacity of graphite with PF_6^- intercalation was reported to be between 93 mAh g^{-1} ($C_{24}PF_6$) and 112 mAh g^{-1} ($C_{20}PF_6$)[1]. Moreover, in our case, the discharge time of our electrodes at 100 mA g^{-1} was close to 1 h (0.98 h for the graphite electrode, 0.96 h for the C2DP-G electrode). At low rates (0.1–3 C), the curves nearly overlap with each other, and only slight voltage polarization (voltage gap between the charge and discharge plateaus) and capacity decay were detected. At high rates (5 C and 10 C), the discharge plateaus, particularly the high-voltage plateau (>4.4 V), become sloped with apparently decreased voltage. This shape change comes from the large voltage polarization (the combination of activation polarization, ohmic polarization, and concentration polarization).

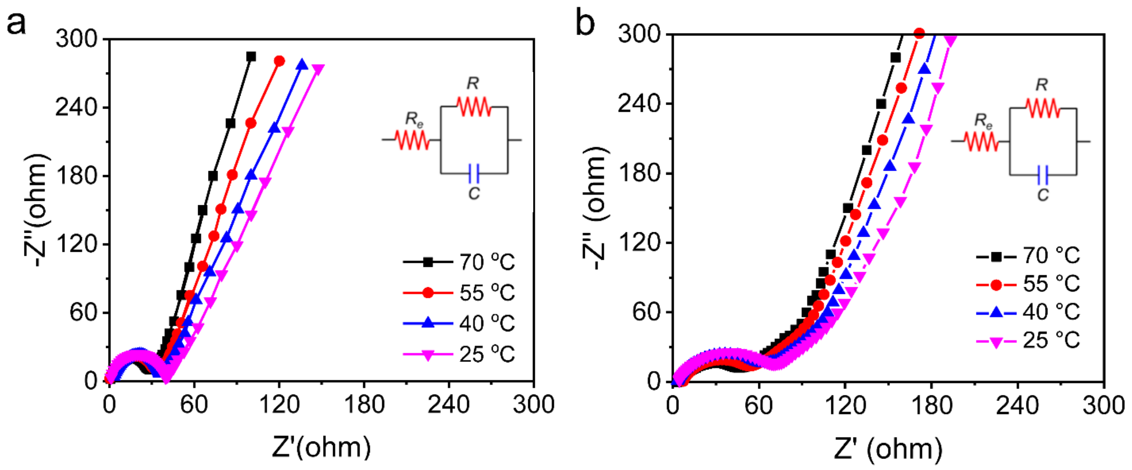

Supplementary Figure 14. Nyquist plots of **a** PP and **b** C2DP-PP at different temperatures. Insets show the equivalent circuit for the EIS fitting.

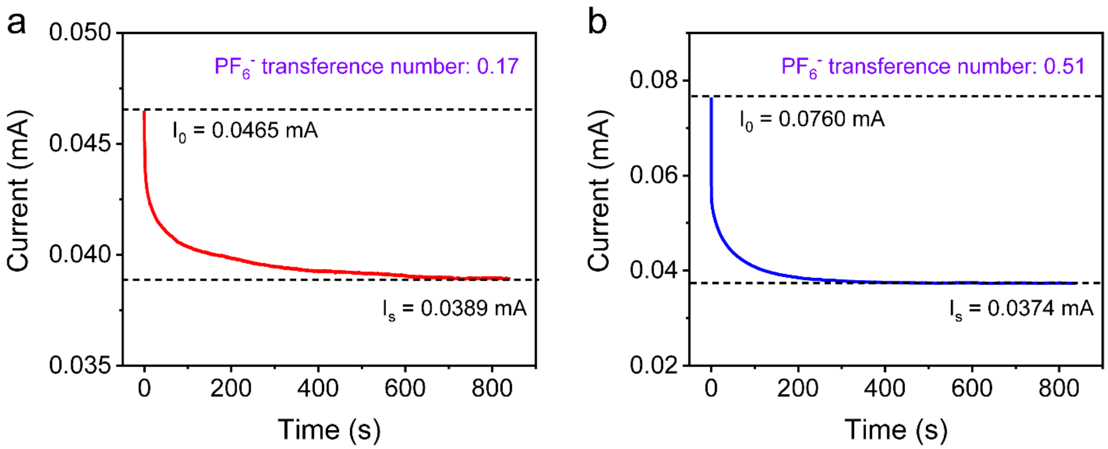

Supplementary Figure 15. Chronoamperometry profiles of **a** PP and **b** C2DP-PP in symmetric Li//Li Swagelock cells with a step potential of 10 mV. I_0 and I_s represent the current at the beginning state and steady state, respectively.

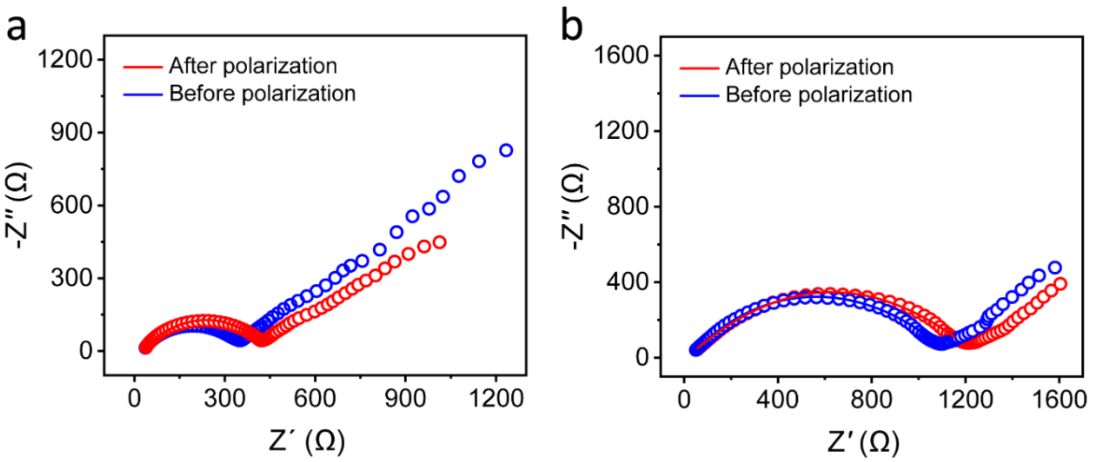

Supplementary Figure 16. Nyquist plots of **a** the Li//PP//Li cell and **b** the Li//C2DP-PP//Li cell. The initial resistance (R_0) and steady-state resistance (R_s) were derived by the EIS measurement of the symmetric cells (*i.e.*, Li//PP//Li and Li//C2DP-PP//Li) before and after polarization, respectively.

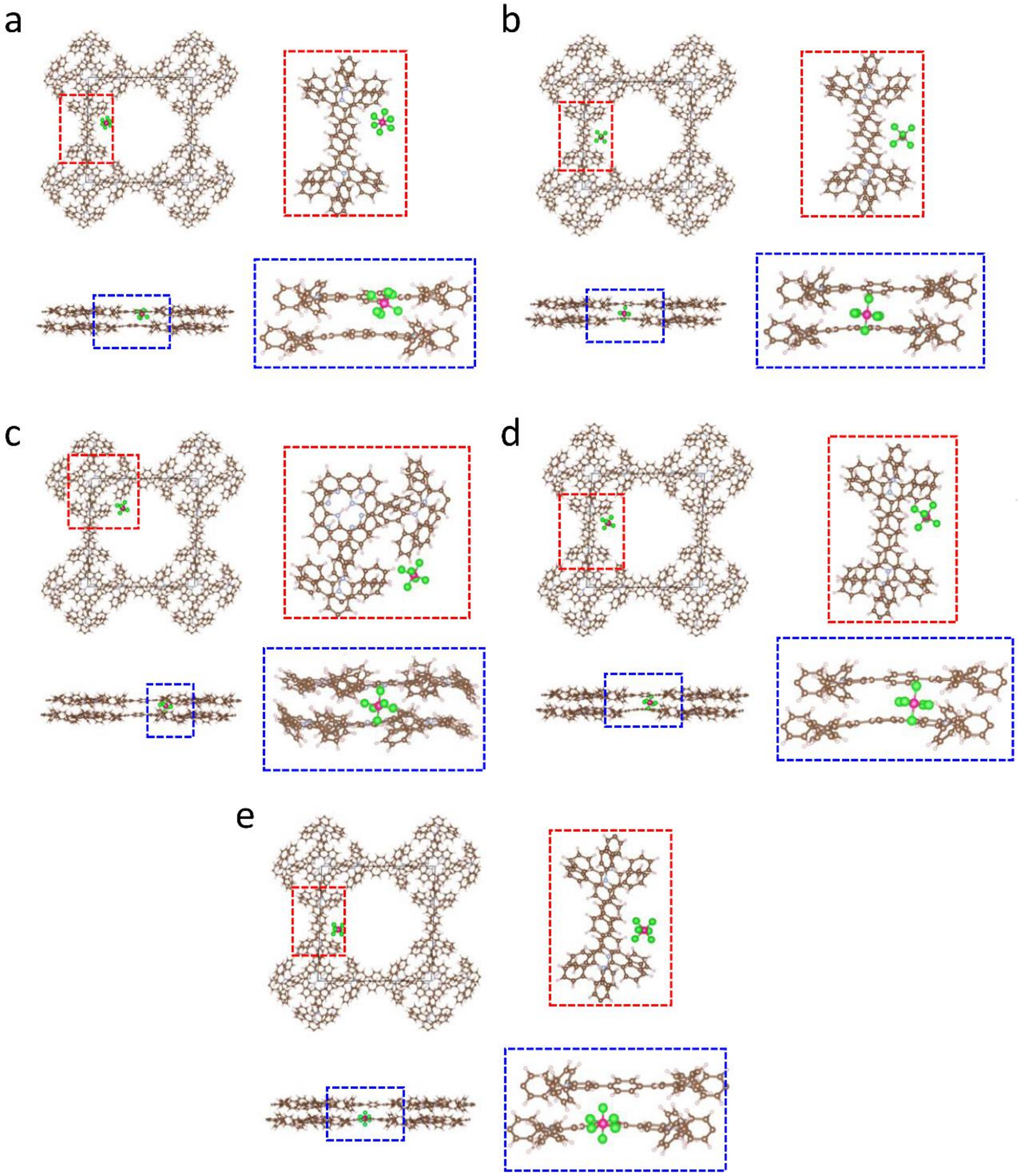

Supplementary Figure 17. Top and side views showing various configurations of C2DP with one PF_6^- anion, including **a** site-1, **b** site-2, **c** site-3, **d** site-4, and **e** site-5. Site-4 was calculated as the most thermodynamically stable configuration.

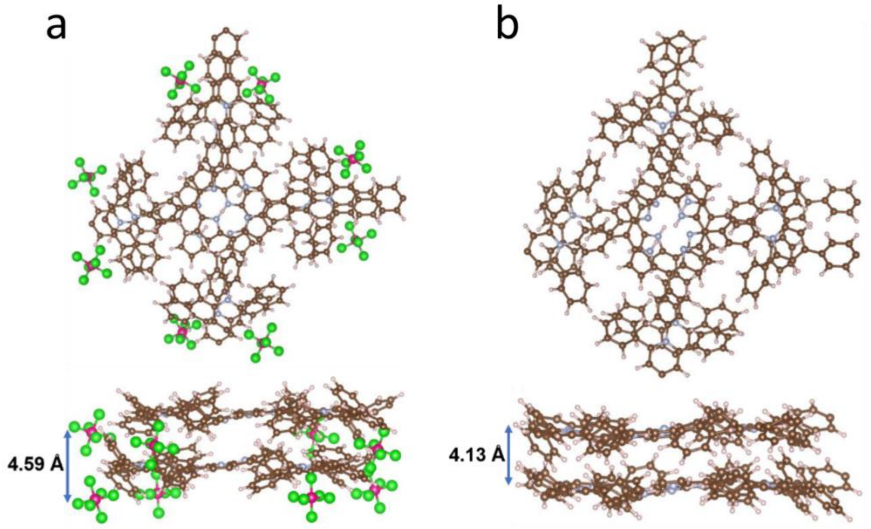

Supplementary Figure 18. Atomic configurations of C2DP **a** with and **b** without charge-compensated PF_6^-. Compared with non-PF_6^- structure (4.13 Å), PF_6^--containing C2DP has a slightly larger interlayer distance of 4.59 Å, supporting the intermolecular electrostatic interaction.

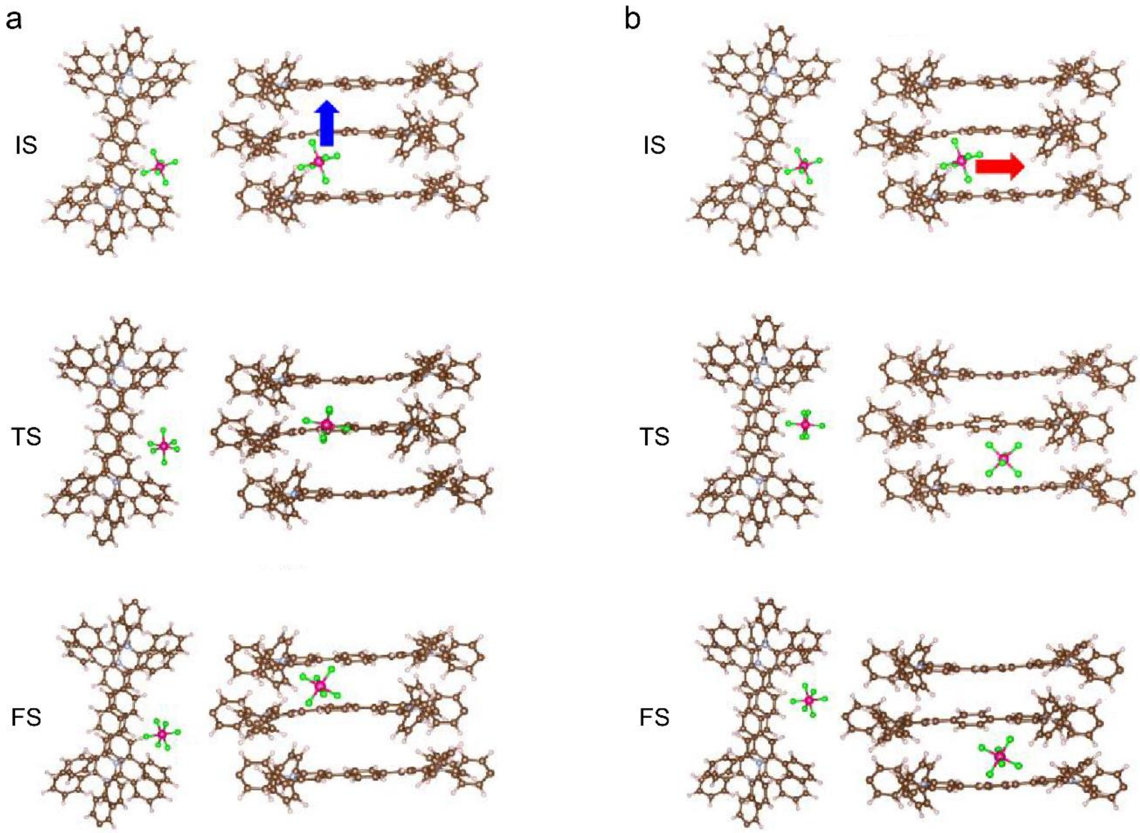

Supplementary Figure 19. Initial state as reactant (IS), transition state (TS) and final state(FS) as products for PF_6^- diffusion in **a** Path-1 and **b** Path-2.

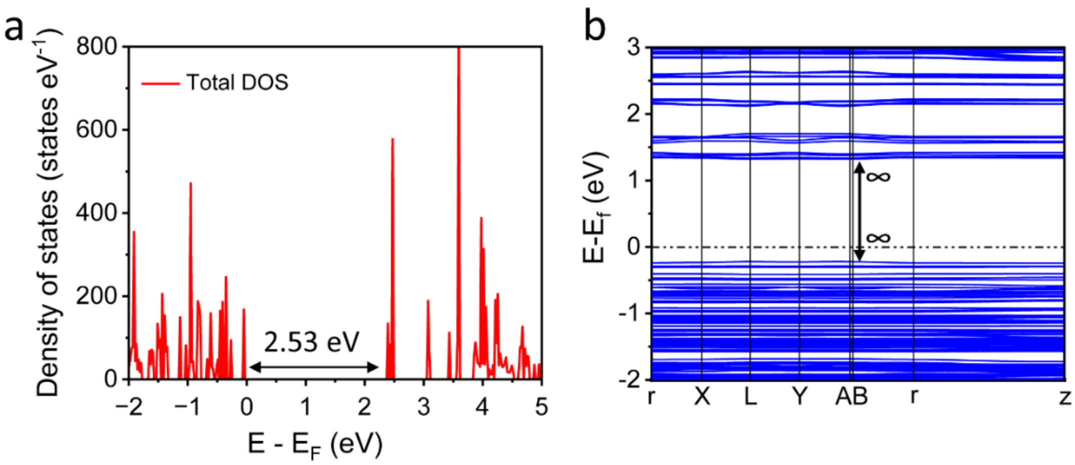

Supplementary Figure 20. **a** The HSE06 DOSs and **b** the PBE band structure of C2DP.

To evaluate the electron-transport property of C2DP, we calculated the HSE06 (Herd–Scuseria–Ernzerhof hybrid) functional density of states (DOSs) and the Perdew-Burke-Ernzerhof band structure of C2DP (Supplementary Figure 20). Here, the computationally expensive HSE06 method provides a comparatively accurate assessment of the band gap, and the PBE band structure can precisely explain the nature of bands. It was revealed that C2DP possessed a direct band gap of 2.53 eV. On a closer look, the band structure consists of all flat bands with no exception for valence band maximum (VBM) and conduction band minimum (CBM). These flat VBM and CBM bands are associated with holes and electrons with infinite effective masses and zero velocity. Thus, the electrons/holes are "localized" or stuck around particular spatial locations, suggesting the very low hole/electron conductivity of C2DP.

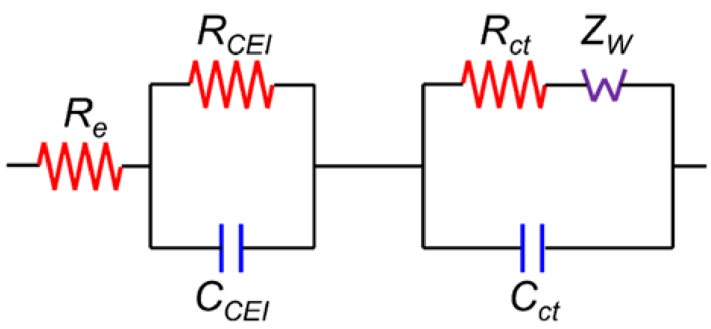

Supplementary Figure 21. Equivalent circuit used for the analysis of the EIS results.

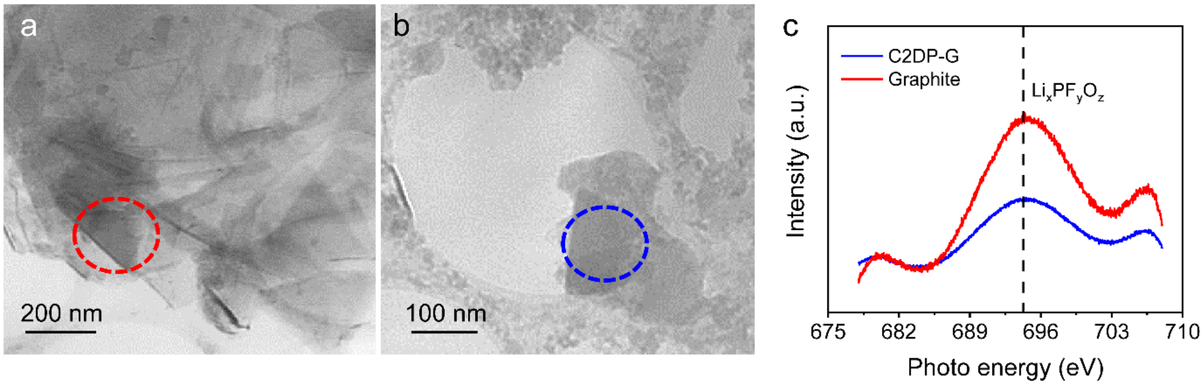

Supplementary Figure 22. Microscopic images and area selection for the XANES measurement of **a** the graphite and **b** the C2DP-G electrode. **b** F K-edge XANES spectra of the graphite electrode and the C2DP-G electrode after 3 GCD cycles at 1C.

Both spectra show a broad peak at around 694.8 eV, which coincides with the $Li_xPF_yO_z$ component from electrolyte decomposition. The content of $Li_xPF_yO_z$ is obviously higher in the graphite electrode than in the C2DP-G electrode, implying the considerable electrolyte decomposition on the graphite electrode.

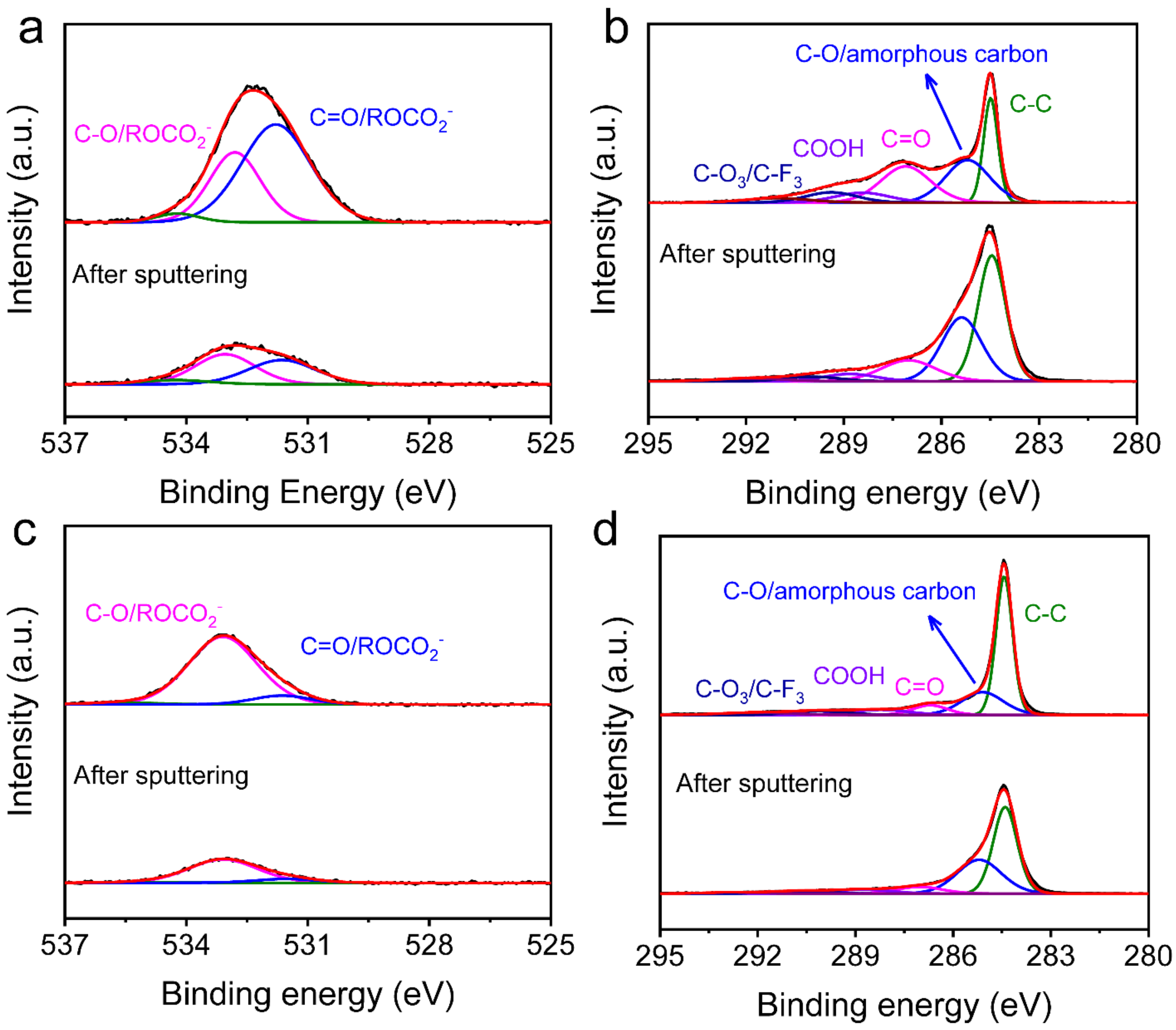

Supplementary Figure 23. **a** O 1s and **b** C 1s XPS spectra of the fully charged graphite electrode. **c** O 1s and **d** C 1s XPS spectra of the fully charged C2DP-G electrode.

In the O1s and C1s XPS spectra of the fully charged C2DP-G electrode, signals associated with O species could come from the used electrode binder (*i.e.*, alginate acid sodium salt) and the carbon black additive. In the C 1s spectra, deconvoluted peaks at 291.5 eV (C-O_3,C-F_3), 288.7 eV (COOH), and 287.9 eV (C=O) can be ascribed to the electrolyte decomposition products.[2] Meanwhile, the peak at 285.3 eV (C-O/amorphous carbon) comes from the combination of the electrolyte decomposition products, the binder, and the carbon black additive. The vanished C=O signal in the C2DP-G electrode evidences the suppressed electrolyte decomposition on the C2DP-G electrode. A similar conclusion can also be drawn by comparing the O 1s spectra of the fully charged graphite electrode and the fully charged C2DP-G electrode[3].

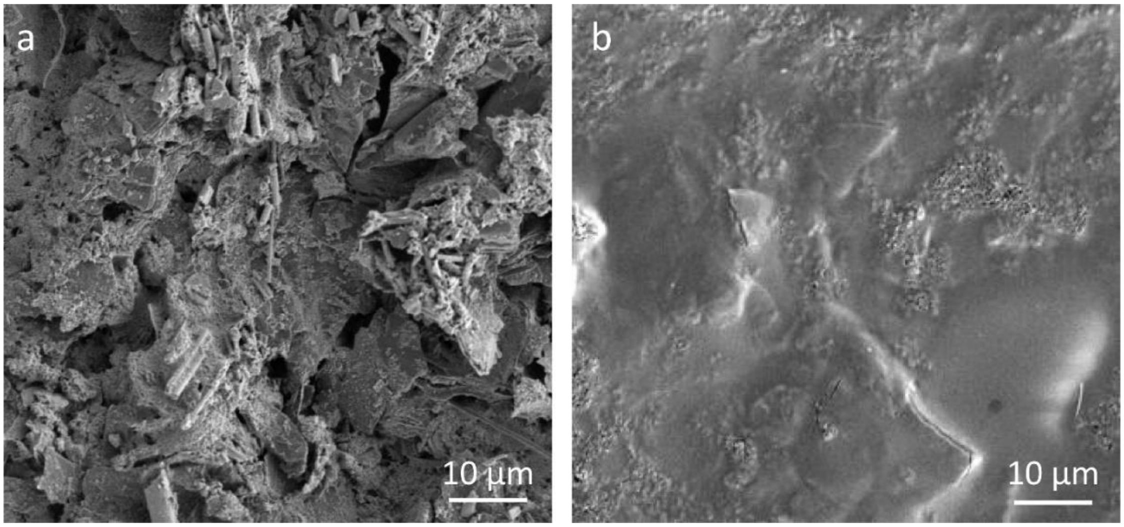

Supplementary Figure 24. SEM images of **a** the graphite electrode and **b** the C2DP-G electrode after 100 GCD cycles. The continuous C2DP layer was clearly identified on the surface of the C2DP-G electrode, supporting the strong robustness of C2DP in the battery environment.

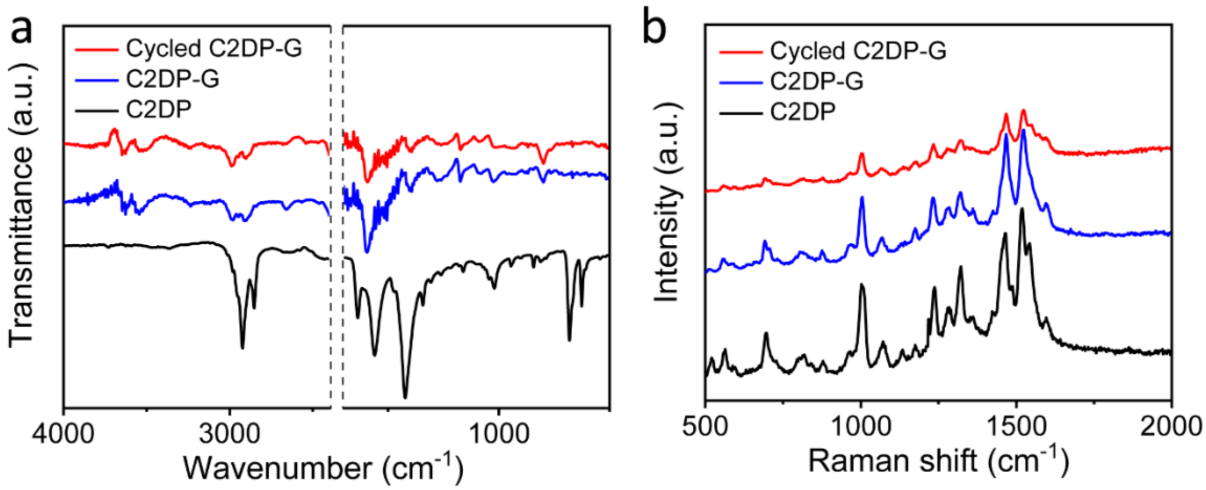

Supplementary Figure 25. a FTIR and **b** Raman spectra of C2DP and the C2DP-G electrode before and after 100 GCD cycles at 2 C. Apparently, the C2DP-G electrode shows almost the same peaks before and after cycling.

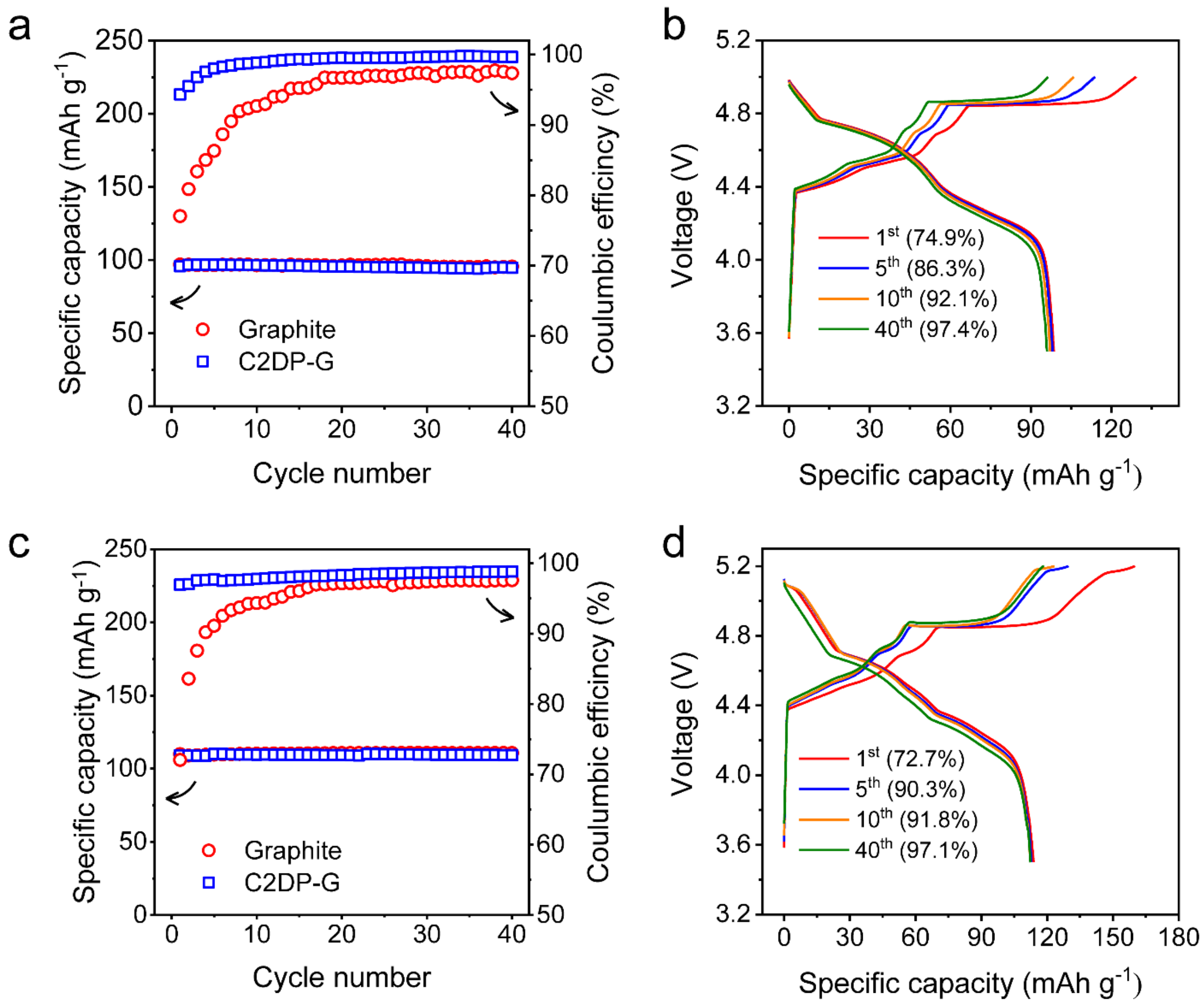

Supplementary Figure 26. **a** Coulombic efficiencies of the graphite electrode and the C2DP-G electrode in 2 M LiFSI. **b** GCD curves of the graphite electrode in 2 M LiFSI. **c** Coulombic efficiencies of the graphite electrode and the C2DP-G electrode in 2 M LiTFSI. **d** GCD curves of the graphite electrode in 2 M LiTFSI.

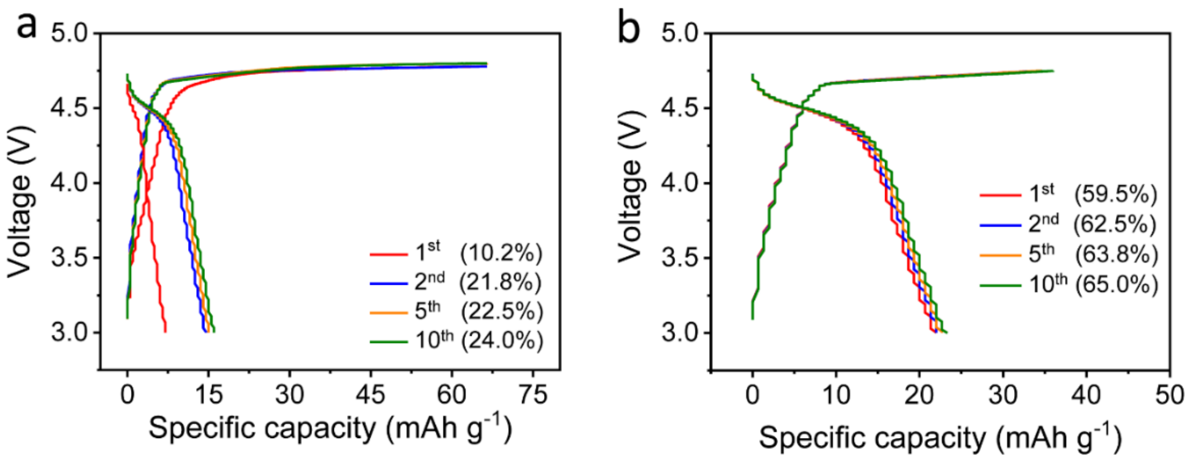

Supplementary Figure 27. The 1st, 2nd, 5th, and 10th GCD profiles of **a** the graphite electrode and the C2DP-G electrode in 2 M $LiClO_4$ dissolved in propylene carbonate at 0.3 C and 50 °C.

We have further studied the graphite electrode and the C2DP-G electrode in an electrolyte of 2 M $LiClO_4$ dissolved in propylene carbonate at 50 °C. In fact, the ClO_4^--intercalation chemistry of graphite was demonstrated to be not reversible and efficient by the early study.[4] At an elevated temperature of 50 °C, ClO_4^- only achieved the stage-V intercalation with a low specific capacity (below 15 mAh g^{-1}) and Coulombic efficiency (below 25%). Supplementary Figure 27 compares the GCD profiles of both electrodes at 0.3 C. Like the early study, the graphite electrode in our case depicted a low specific capacity of 7 mAh g^{-1} and low Coulombic efficiencies of 10.2% at the initial cycle and 24% at the 10th cycle. By contrast, the C2DP-G electrode reached a higher specific capacity of 21 mAh g^{-1} and enhanced Coulombic efficiencies of 59.5% at the initial cycle and 65.0% at the 10th cycle.

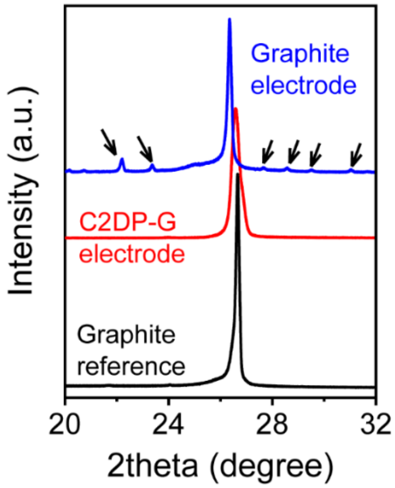

Supplementary Figure 28. XRD patterns of the fully discharged graphite electrode and the fully discharged C2DP-G electrode after 10 GCD cycles at 0.3 C in 2 M $LiClO_4$ dissolved in propylene carbonate and 50 °C.

The XRD patterns were collected for the discharged electrodes after 10 GCD cycles at 0.3 C in 2 M $LiClO_4$ dissolved in propylene carbonate and 50 °C (Supplementary Figure 28). Several side peaks beside the (002) plane were detected in the graphite electrode, which can be assigned to confinement of the aggregated intercalation compounds (*i.e.*, complexes of Li^+, ClO_4^-, and propylene carbonate) between the graphite layers. However, these side peaks were not observed in the C2DP-G electrode.

Supplementary Table 1. The R_0 and R_s values of Li//PP//Li and Li//C2DP-PP//Li.

**Type of cell**	$\boldsymbol{R_0}$	$\boldsymbol{R_s}$
**Li//PP//Li**	498 Ω	401 Ω
**Li//C2DP-PP//Li**	1230 Ω	1158 Ω

Supplementary Table 2. The EIS fitting result of the graphite electrode and the C2DP-G electrode before and after 20 GCD cycles.

**Electrode**	**Electrolyte resistance (R_e)**	**Interphase resistance (R_{CEI})**	**Charge-transfer resistance (R_{ct})**
**Graphite before cycling**	2.7 Ω	7.4 Ω	98.5 Ω
**Graphite after 20 cycles**	5.8 Ω	93.5 Ω	142.6 Ω
**C2DP-G before cycling**	1.8 Ω	10.7 Ω	148.6 Ω
**C2DP-G after 20 cycles**	3.4 Ω	17.3 Ω	101.3 Ω

Supplementary Table 3. Durability of anion-intercalation chemistries of graphite.

Anion type/ molarity	Current density	Initial Coulombic efficiency	Initial specific capacity	Cycling stability	Ref
FSI^-/ 5 M	100 mA g^{-1}	85%	98 mAh g^{-1}	87% capacity retention after 300 cycles	5
PF_6^-/ 2 M	100 mA g^{-1}	87.5%	95.1 mAh g^{-1}	96% capacity retention after 50 cycles	6
PF_6^-/ 1M	200 mA g^{-1}	Pristin graphite: 60%	70 mAh g^{-1}	22% capacity retention after 150 cycles	2
	200 mA g^{-1}	SEI-graphite: 49%	82 mAh g^{-1}	96% capacity retention after 500 cycles	
$TFSI^-$/ 3 M	100 mA g^{-1}	93%	117 mAh g^{-1}	96.2% retention after 50 cycles	7
FSI^-/ 7.5 M	200 mA g^{-1}	91.5%	97 mAh g^{-1}	96% capacity retention after 500 cycles	8
PF_6^-/ 4 M	200 mA g^{-1}	67%	105 mAh g^{-1}	88% capacity retention after 200 cycles	9
PF_6^-/ 3 M	100 mA g^{-1}	58%	120 mAh g^{-1}	94% capacity retention after 300 cycles	10
PF_6^-/ 2 M	100 mA g^{-1}	92%	98 mAh g^{-1}	92.8% capacity retention after 1000 cycles	**Our work**
$TFSI^-$/ 2 M	100 mA g^{-1}	92.2%	119 mAh g^{-1}	88.7% capacity retention after 1000 cycles	
FSI^-/ 2 M	100 mA g^{-1}	94.2%	99 mAh g^{-1}	89.1% capacity retention after 1000 cycles	

Supplementary Reference:

1. Read, J. A. In-situ studies on the electrochemical intercalation of hexafluorophosphate anion in graphite with selective cointercalation of solvent. *J. Phys. Chem. C* **119**, 8438–8446 (2015).

2. Li, W. H. *et al.* Highly Improved Cycling Stability of Anion De-/Intercalation in the Graphite Cathode for Dual-Ion Batteries. *Adv. Mater.* **31**, 1804766 (2019).

3. Han, X. *et al.* An In Situ Interface Reinforcement Strategy Achieving Long Cycle Performance of Dual-Ion Batteries. *Adv. Energy Mater.* **9**, 1804022 (2019).

4. Gao, J., Tian, S., Qi, L. & Wang, H. Intercalation manners of perchlorate anion into graphite electrode from organic solutions. *Electrochim. Acta* **176**, 22–27 (2015).

5. Kravchyk, K. V. *et al.* High-energy-density dual-ion battery for stationary storage of electricity using concentrated potassium fluorosulfonylimide. *Nat. Commun.* **9**, 4469 (2018).

6. Wang, G. *et al.* Polarity-Switchable Symmetric Graphite Batteries with High Energy and High Power Densities. *Adv. Mater.* **30**, 1802949 (2018).

7. Wang, G. *et al.* An Anode-free Zn-graphite Battery. *Adv. Mater.* **12**, 2201957 (2022).

8. Xiang, L. *et al.* Highly Concentrated Electrolyte towards Enhanced Energy Density and Cycling Life of Dual-Ion Battery. *Angew. Chemie - Int. Ed.* **59**, 17924–17930 (2020).

9. Zhang, X., Tang, Y., Zhang, F. & Lee, C. A Novel Aluminum – Graphite Dual-Ion Battery. *Adv. Energy Mater.* **6**, 1502588 (2016).

10. Wang, S. *et al.* A Novel Ultrafast Rechargeable Multi-Ions Battery. *Adv. Mater.* **29**, 1606349 (2017).
